# Fused Microknot Optical Resonators in Folded Photonic Tapers for in-Liquid Durable Sensing

**DOI:** 10.3390/s18051352

**Published:** 2018-04-26

**Authors:** Alexandra Logvinova, Shir Shahal, Moti Fridman, Yoav Linzon

**Affiliations:** 1 School of Mechanical Engineering, Faculty of Engineering, Tel Aviv University, Tel Aviv 69978, Israel; 2 Faculty of Engineering and the Institute of Nanotechnology and Advanced Materials, Bar-Ilan University, Ramat Gan 5290002, Israel; shirshahal@gmail.com (S.S.); mordechai.fridman@biu.ac.il (M.F.)

**Keywords:** optical resonators, optical sensing, liquids sensors

## Abstract

Optical microknot fibers (OMFs) serve as localized devices, where photonic resonances (PRs) enable self-interfering elements sensitive to their environment. However, typical fragility and drifting of the knot severely limit the performance and durability of microknots as sensors in aqueous settings. Herein we present the fabrication, electrical fusing, preparation, and persistent detection of volatile liquids in multiple wetting–dewetting cycles of volatile compounds and quantify the persistent phase shifts with a simple model relating to the ambient liquid, enabling durable in-liquid sensing employing OMF PRs.

## 1. Introduction and Overview

Miniaturized active devices operated in liquid media are an attractive venue for localized sensing, potentially offering precise, low-cost, rapid, and sensitive environmental detection. Various approaches include optical-fiber [1,2,3,4,5], micro-mechanical [6,7,8], and plasmonic-nanowire [9] based environmental sensing in aqueous media. In particular, micro-structured fiber-optic transmission devices used as optics sensors offer comparative advantages over the alternative approaches (see, e.g., a recent review and references therein [1]). In particular, optical microknot fibers (OMFs) are a promising platform in which the photonic resonance can be exploited [10]. Even though fiber tapers [11,12], rings [13], and knots [14] were used in previous research as sensors of refractive index [15,16], temperature [17,18], and force [19,20], the durability and sustainability in microknot geometry has been limited to few cycles of operation. In fiber-based photonic resonators, despite their natural fragility [1], knot geometries are superior in some aspects as compared to ring geometries [2]. Photonic resonances based on ring geometries can be based on critical coupling adjusted by mechanical positioning via expensive piezoelectric actuators [21], or can be fixed lithographically [22], enabling high-*Q* resonances sensitive to the environment [23]. Meanwhile, the manually prepared OMF geometry is self-aligned and can be tuned in diameter by pulling [24]; however, this geometry is mechanically fragile [25] and exhibits relatively low *Q* values. Thus, the simplicity of OMFs as sensors based on photonic transmission is typically accompanied by degraded performance as well as mechanical fragility. The sustainability of OMFs can be significantly enhanced [26] based on fusing manually-prepared OMFs around a desirable knot diameter, either by electrical [27,28] or other [29,30,31] techniques.

In this paper, we present and test two preparation methods that are both repeatable and durable over long operation, as opposed to previous fragile implementations relating to OMF operation in liquid environment. Specifically, in the case of folded photonic tapers that incorporate microknot resonators, improved operation in various aqueous environments has been demonstrated. We also quantify the OMF spectral characteristics in principal component (PC) space, and find unique PC-space recognition plots, both in pure and mixed ambient liquids.

## 2. Experimental Details

**Figure 1:**
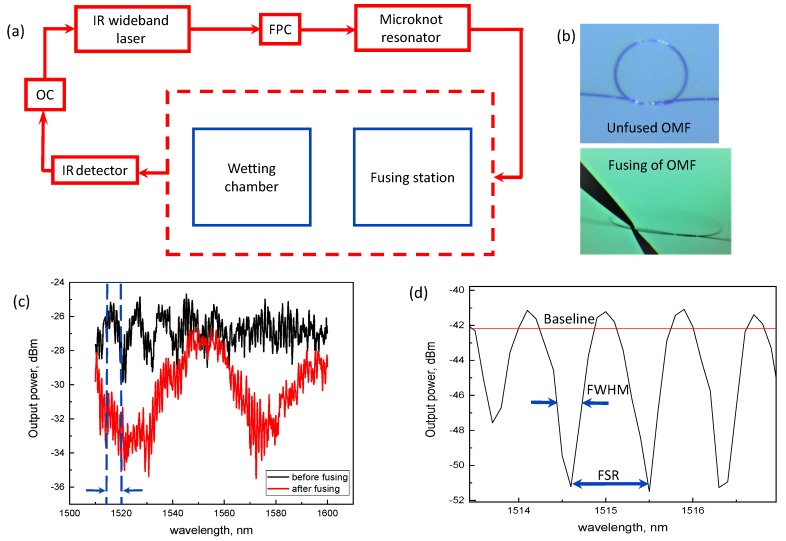
Basic experimental details. (**a**) Setup schematics; (**b**) Photographs of optical microknot fiber (OMF) before fusing (above) and during fusing (below); (**c**) Broad spectrum of OMF corresponding to the conditions in panel (**b**). Blue lines are the region of interest displayed in Figure 1d; (**d**) Magnified OMF spectrum within a narrow band. FPC: polarization controller; FSR: free spectral range; FWHM: full width at half maximum; IR: infrared; OC: operating computer.

The first version of the in-liquid optical sensor is based on fused optical microknot resonators defined locally by manual preparation on optical fiber tapers. Tapers of high uniformity (5–30 mm in length, 3–7 µm in waist diameter) were produced on the basis of single-mode low-loss fused silica fibers (single-mode fiber, SMF; wavelengths of operation 1.5–1.6 µm) using an AFL Lazermaster LZM-100 splicing system [25,26]. OMFs were produced manually by winding an optical fiber into a knot, followed by pulling to minimum diameters (ranging 0.5–1 mm). The OMF thus obtained was coupled to a wideband infrared (IR) laser and subjected to local electrical fusing following the procedure described previously [26]. An illustration of the experiment is shown in Figure 1.

Figure 1a shows schematics of our experimental setup including a fusing probe-station and wetting chamber, where OC is the operating computer and FPC is a polarization controller. Figure 1b shows a magnified image of the microknot before fusing and together with a pair of probes introduced within the coupling area. The transmission spectra, in logarithmic scale, of the fused (red line) and unfused (black line) OMFs are presented in Figure 1c. The periodic OMF resonances enable real-time experimental determination of the free spectral range (FSR), full width at half maximum (FWHM), quality factor (*Q*), phase shift (PS) and baseline (BL) (see Figure 1d). The in-liquid sensing properties of OMFs were investigated at room temperature in the presence of five pure liquids (ethanol, methanol, isopropanol, acetone, distilled water, all 95% absolute) and two 50% mixtures (ethanol/methanol (ETM) and isopropanol/deionized (DI) water(DII)). In all experiments, the same device incorporating a taper of 7 µm waist and a microknot of approximately 0.5 mm diameter was used.

Both straight (mounted) and folded geometries can be useful in different conditions. For example, the former is useful in the context of localized packaged detection with the medium delivered into the package, and the latter in the context of remote sensing, where the OMF is brought to an exposure medium. We emphasize that the straight case was tested with sub-optimal mounting conditions, where a nearby glass substrate was used. With the use of either a hydrophobic substrate (such as a flat PDMS surface) or a raised MKR taper, the instability problem encountered with straight configuration geometry would certainly be overcome. Therefore, our comparison of the straight and folded geometries presented below is not symmetrical or straightforward.

**Figure 2:**
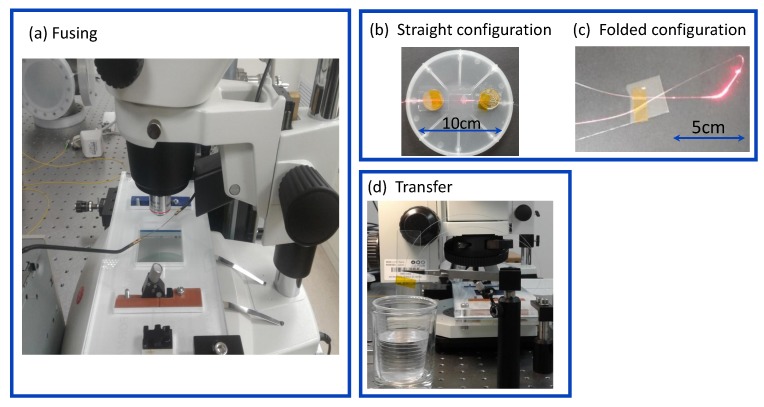
Stages of OMF preparation. (**a**) Fusing; (**b**) Straight configuration following positioning; (**c**) Folded configuration following positioning; (**d**) Transfer to measurement chamber.

The details of OMF preparation are presented in Figure 2. A photograph given in Figure 2a shows a microscope station with introduced electrical probes employed in the electrical fusing procedure. We present two OMF preparation methods referred to here as straight (Figure 2b) and folded (Figure 2c) OMF configurations. In the former case, the OMF was attached to a glass substrate containing a slot where the OMF center was located. Then, an OMF clamped to a glass slide was placed inside a wetting vessel and fixed to prevent its floating after injection and drying of the liquid. As a simplified alternative, a folded configuration included the OMF wound to a loop and attached to a glass substrate, as shown in Figure 2c. Figure 2d shows a photograph of a folded OMF placed inside the wetting chamber, still functioning without degradation after 3 months following its preparation. In both straight and folded configurations, a fused OMF was incorporated. For clarity, photos in Figure 2b,c were taken while coupling OMFs to a red laser source emitting at wavelength λ = 660 nm. In both configurations, maximum radiation corresponded to a microknot location, as expected from the segments including the highest curvature in the fiber.

To quantify OMF characteristics as an in-liquid durable sensor, we performed wetting–drying experiments on both pure liquids and binary mixtures. In both straight and folded configurations, we monitored the spectral response of OMFs by coupling its input to a wide-band tunable IR laser source. The input source power was set as constant (0.2 mW) at each point-by-point wavelength setting. The output power was measured using a built-in IR detector having a resolution 0.1 nm (see Figure 1).

## 3. Results and Discussion

**Figure 3:**
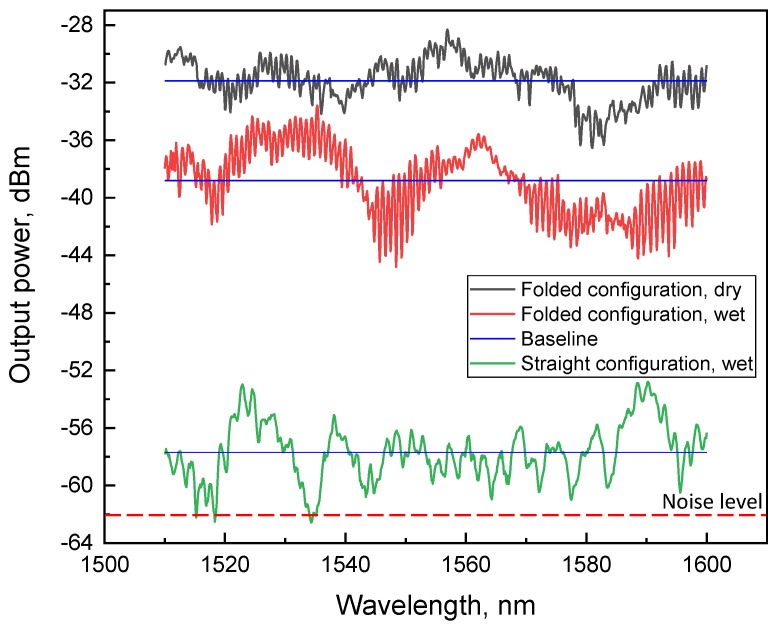
Comparison of transmission spectra in straight and folded configurations. Blue lines are the baselines corresponding to dry and wet states of both straight and folded configurations.

First we considered the repeatability and reversibility properties of straight versus folded configurations under cyclic wetting and drying conditions. Using the same analytes in both configurations (Figure 2b,c), we tracked the OMF transmission spectra (namely the BL level measured) as a real-time indicator of the reversibility. The baseline is defined here as an average transmission. In Figure 3, transmission spectra of both straight and folded configurations are shown. We note that the overall shape of the broad spectrum (including the number of modes and changing coupling strength and spectral envelope) can vary significantly along the entire spectral range within the wetting–dewetting cycles and exposure to different liquids, but within our quantitative analysis, we focus locally on adjacent resonances, within a narrow band (Figure 1d), and monitor the BL, FWHM, FSR, and PS parameters only, assuming simple physics involved (see Equations (1)–(3) below).

The black line shows the spectrum measured in the folded configuration with BL = −32 dBm in ambient air, representing a total transmission loss of −25 dBm as compared to the input power of −7 dBm. Upon wetting with an analyte, a significant deterioration in BL level to approximately −40 dBm was observed (Figure 3, red line). Following dewetting, the BL fully recovered to −32 dBm. Reversibility remained sustainable over approximately 50 cycles of operation and did not demonstrate any further degradation in loss during three months after the original OMF preparation.

In sharp contrast, most cases of the straight configuration resulted in subsequent transmission drops in the noise level after a few dewetting cycles (Figure 3, green line), and did not show the reversibility effect required for durable sensing. The noise level was −62 dBm which was recognized by the detector as power *P* = 0. It is worth noting that this result was observed in all types of liquids we used. To attain reproducibility in this case, prevention of OMF from getting stuck to the bottom surface of the vessel resulting from water cohesion forces is critical, and can be overcome with a choice of a hydrophobic substrate below the OMF. The latter is practically more challenging than the folded configuration, in which the transfer stage is more straightforward and does not require a bottom substrate. We suggest that the two geometries are independently useful in different conditions—for example, for the folded case in remote sensing where the microknot is to be brought to an exposed medium, and the packaged geometry where the medium is brought to the fixed microknot in the package.

**Figure 4:**
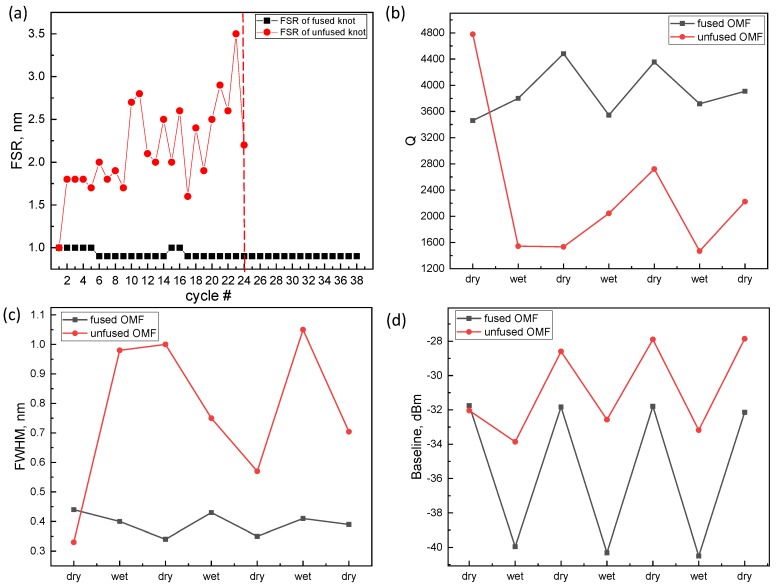
Comparison of derived parameters in fused (black rectangles) and unfused (red circles) OMFs as a function of wetting/drying cycles in ethanol. (**a**) Free spectral range. Breaking point is illustrated by the red dashed line, (**b**) Quality factor; (**c**) Full width at half maximum; (**d**) Baseline.

From the output transmission spectra, focused on a short spectral range of approximately five resonances, we derived high-resolution values for the parameters from the photonic resonances, FSR, FWHM, *Q*, and BL. The measured values are shown in Figure 4 as functions of the wetting–dewetting cycles in both unfused (Figure 1b) and fused as well as folded (Figure 2c) OMFs. Being related to the OMF size [25], FSR relates to stability of its diameter and circular profile. As observed in Figure 4a, the FSR of the fused OMF remained consistently stable over 38 wetting-dewetting cycles, while in unfused OMFs the FSR showed a gradual increase followed by an abrupt fiber breakage as FSR (OMF diameter) surpassed a critical value. Thus, it is evident that electrical fusing provided OMFs with mechanical stability, which is crucial for persistent detection of OMF parameters in multiple in-liquid wetting–drying cycles. Figure 4b–d show data for FWHM, *Q*, and BL monitoring for unfused (red circles) and fused (black rectangles) OMFs.

We found that in fused OMFs, a linear switching of all three parameters occurred between dry and wet states. Moreover, the transition in BL was consistently reproduced after each wetting–dewetting cycle and demonstrated stable switching in prolonged operation (up to three months tested).

In contrast, unfused OMFs showed a more than threefold degradation of *Q* after the first wetting, which did not restore to the initial level, exhibiting arbitrary fluctuations during wetting–dewetting stages. A parallel BL switching effect was also observed in the unfused OMF, which did not possess repeatability in FWHM or in *Q*-factor. This result further corroborates our conclusion that in-liquid durable sensing with OMFs is viable only with a combination of both fusing and subsequent folded configuration used.

**Figure 5:**
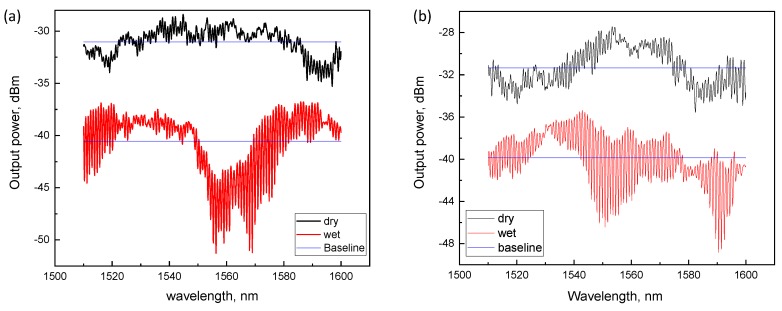
Transmission spectra of OMF in different volatile mixtures: (**a**) ETM (**b**) DII. Blue lines represent the baselines corresponding to dry and wet conditions for each mixture.

Along with pure liquids, we analyzed OMF operation in binary mixtures of volatile liquids: ethanol + methanol (denoted ETM) and isopropanol + DI water (denoted DII), in 50:50% ratio by volume. Transmission spectra in OMF (fused and folded cases) both before and after immersion in ETM are displayed in Figure 5a,b, showing the corresponding spectra of the same OMF sensor before and after placement in DII. The same parameters (Figure 1d) were also deduced for mixtures.

**Figure 6:**
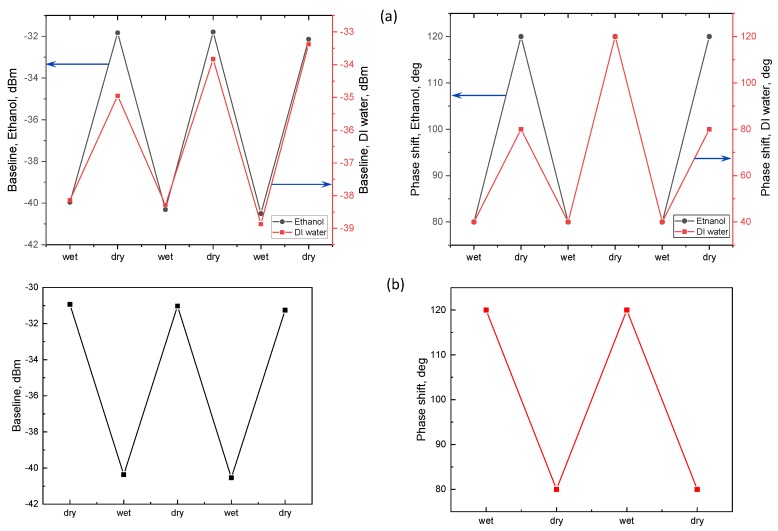
Fused and folded OMF exposed to subsequent wetting–drying cycles. Data series on ethanol, DI water, and 50% mixture of ethanol and methanol (ETM). Baseline and Phase shift as a functions of wetting/dewetting stage. (**a**) Pure liquids; (**b**) ETM mixture.

Spectra from fused OMFs exposed to liquids were phase shifted with respect to the dry condition, and as an additional OMF characteristic, the phase shift (PS) can be experimentally derived from the output transmission spectra, similarly to the other parameters (BL, FSR, FWHM, *Q*). In particular, the PS of the same resonance peak not overlapping beyond the FSR with the subsequent resonance of the OMF directly relates to its surrounding physical environment [1]. To characterize OMF sensing properties, we performed phase shift and baseline monitoring on both pure analytes and mixtures. Figure 6a illustrates the OMF responses for pure ethanol (black line) and DI water (red line). The PS and baseline are presented as functions of wetting–drying stages. As is evident, the OMF placed in pure ethanol possessed a good repeatability and no degradation in both BL and PS during several cycles. PS was found to maintain constant values switching from 80° in the wet state (with respect to the original unshifted spectrum corresponding to dry state) up to 180° in the subsequent dry state. We found that isopropanol and methanol demonstrated the same behavior, whereas in presence of acetone the OMF spectral characteristics deteriorated within 2–3 wetting–dewetting cycles. This can be explained by the fact that the electrical fusing—pertinent to the stability of the microknot—includes residues of heated aluminum and organic compounds resulting from the tips’ contamination. These could be dissolved in acetone, which is a well-known solvent for organic compounds [32]. DI water also demonstrated a reversible transition between wet and dry states, producing a similar switching pattern (Figure 6a). An OMF response for the

ETM mixture is shown in Figure 6b and again shows a good reproducibility for both BL and PS, noticing the fact that the BL peak-to-peak values remained the same, and the PS switched repeatably between +120° and 80° within multiple cycles, with the wet PS now being larger than in the dry case.

Physical interpretation can be given to the observed results with a model relating the measured OMF parameters with the physical and chemical environment [1]. We again emphasize that in the broad spectrum measured in the experiment there can be strong variability in parameters such as coupling strength, total number of modes, and spectral envelope, between dry and wet conditions, involving more complex physics, but here we emphasize the analysis pertaining to nearby resonances in a magnified spectrum around a chosen central wavelength (as in Figure 1d). Of particular relevance are changes in refractive index (*n*) and temperature (*T*) within the OMF vicinity [15,16,17,33,34], and secondary effects can be due to chemical composition [14,35]. All effects are accumulated in the measured PS, defined as:

(1) $$PS=\frac{\Delta \lambda }{\lambda_{r}}<\frac{FSR}{\lambda_{r}},$$

where $\lambda_{r}=\lambda_{0}/n$ is the original resonance wavelength in medium of refractive index *n*, $\lambda_{0}$ is the vacuum wavelength, and Δλ is the observed wavelength shift of the same resonance not exceeding the FSR. Within the simplest 1D model for the OMF, assuming single-mode propagation of the radiation field, a simple expression for the FSR reads [17]:

(2) $$FSR≅\frac{\lambda_{0}^{2}}{n_{g}L},$$

where $n_{g}$ is the group index of the propagation mode within the OMF, and L=πD is the OMF circumference of diameter *D*. Upon changes of ambient index, temperature, or both, the total phase shift resulting from small length and index variations can be approximately estimated from the derivative of Equation (2), assuming the the FSR does not change, plugged into Equation (1) [17]:

(3) $$PS≅\frac{\Delta L}{L}+\frac{\Delta n}{n}.$$

Thus, the phase shift, to a first approximation, is an estimate of the combined relative index and OMF length changes (the latter, for example, resulting from thermal expansion).

**Figure 7:**
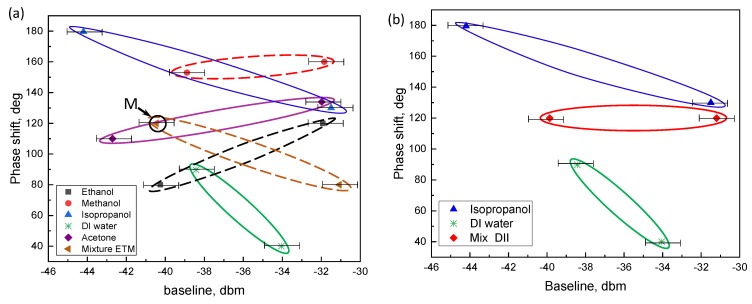
Recognition plots in parameter space. (**a**) Pure liquids as well as ETM mixture (the latter denoted as M); (**b**) isopropanol, DI water, and DII compound plotted separately.

In favor of the unique recognition of ambient liquid, and based on the experimentally determined spectral quantities, we defined 2D principal component space for both pure analytes and mixed liquids, where baseline and PS were chosen as the principal components. For each analyte (including both pure volatile liquids and mixtures), the averaged phase shift and baseline were measured, followed by averaging performed over three data series corresponding to wet and dry OMF states separately. Thus, we obtained two averaged characteristics describing wet and dry conditions respectively, which were plotted in PS–BL parameter space. Data points for the recognition plots (enclosed by ellipses for clarity) corresponding to each liquid of interest are shown in Figure 7, and all data are summarized in Table 1 below. Horizontal error bars in the BL values were estimated by taking the maximum transmission of the spectrum in the spectral range of the central resonance, as well as the minimum transmission observed within this range, and defining the dynamical range as (maximum transmission) − (minimum transmission), and the visibility as the dynamical range/(maximum transmission) − (minimum transmission) (see Figure 5). The error bars in the BL were therefore defined as the +/− DR/2, subsequently in all data presenting the BL. Other parameters (FSR, *Q*, FWHM, and PS) were derived directly from the wavelength scale of the spectrum, and their error was negligible, as opposed to the BL estimation. In mixtures, it was found that at least one of the averaged phase shifts measured was located consistently on the line in between the corresponding PS of its constituent components in Figure 7b. Figure 7a shows this result, where the point designated as *M* corresponds to the averaged wet PS of the OMF exposed to ETM wetting. We therefore conclude that even in remote sensing where a distant folded OMF is in an unknown liquid, operation in different volatiles within these five calibrated volatiles, as well as their mixtures, could be deduced from the OMF transmission resonances parameters.

Finally, as in any sensor, and to serve as a comparative basis with other platforms, it is important to estimate the detection capabilities in our experiment, in terms of the measurements’ resolution, sensitivity, and limit of detection (LOD). Firstly, the resolution of the horizontal wavelength setpoint is given by the IR laser filter specifications as δλ = 0.01 nm = 10 pm. Here δ denotes error, in the resolutions’ case pertaining to the instruments’ capabilities. Similarly, the resolution of the power measured at the fiber output (vertical dimension in Figure 1d, Figure 3, and Figure 5) were determined by the IR detector head used in the experiment, given in linear scale as δP = 0.1 nW = 100 pW detection, or in the logarithmic scale as −70 dBm. The transmission measured is relative to the fixed input power value of $P_{in}$ = 200 µW = 0.2 mW, or −7 dBm injected by the laser. Next, using these instrument-determined resolutions, we can estimate the sensitivities of the calculated quantities in our experiments, quantifying the sensor, determined by Equations (1) and (2). For the phase shift we can differentiate Equation (1) to read $\delta \left(PS\right)=\delta \left(\Delta \lambda /\lambda_{0}\right)=\left(\delta \left(\Delta \lambda \right)\lambda_{0}-\Delta \lambda \delta \left(\lambda_{0}\right)\right)/\lambda_{0}^{2}$≃ 0.01/1520 rad = 6.5 µrad, as an estimate of the minimum detectable phase shift. Similarly, for the FSR, and assuming no errors in either length or index estimations, we can differentiate Equation (2) to read $\delta \left(FSR\right)=2\lambda_{0}\delta \left(\lambda_{0}\right)/n_{g}L$≃ 1520 × 0.01 / $10^{5}$ = 1.52×$10^{-4}$ nm = 0.152 pm. As for the baseline, it is a direct measure of the power level, and thus its sensitivity is equal to the power detection resolution given above: δ(BL)=δ(P) = 100 pW. Using these ultimate instrument resolutions and estimated sensitivities of calculated quantities, one could estimate the ultimate limits of detection in a physical quantity. For example, in estimating the refractive index change in dimensionless refractive index units (RIU), related to the phase shift through Equation (3), and assuming zero change in the total MKR length, we could estimate: $LOD\left(\Delta n,\Delta L=0\right)=\delta \left(PS\right)n_{g}≃6.5\times 10^{-6}\times 1.35$ = 8.8 $\times 10^{-6}$ RIU.

## 4. Conclusions

In this paper, we presented a durable in-liquid sensing approach incorporating fused OMF in folded photonic tapers. The improved folded configuration was thoroughly compared to the typical straight configuration. Local electrical fusing, providing mechanical strengths and good optical performances, enhanced the sustainability of the OMFs and enabled their persistent sensing over long operation including many wetting–dewetting cycles. Fused OMFs demonstrated superior repeatability as opposed to unfused OMFs that were strongly dependent on the ambient conditions with unpredictable behavior and drift with time. This configuration showed good reproducibility and good sensing properties. Unique recognition plots in principal components space specific to five volatile liquids were found, and it was shown that measurements in non-abrasive volatile liquids and mixtures are possible with plausible recognition probability. This suggests that the OMFs operating in both fused and folded configuration, trained in principal component space, can also be operated in remote sensing mode, where a folded OMF connected to a long optical fiber is sent into an unknown distant environment—which could also include mixtures of the calibrated environments—and recognized correctly. This simple and robust OMF configuration can be used in next-generation in-liquid durable sensors.

## Figures and Tables

**Table 1:** Summary of data relevant to the defined principal components as extracted from the experiments.

Liquid	RI	Baseline, Wet State	Baseline, Dry State	Phase Shift, Wet State	Phase Shift, Dry State
Ethanol	1.36	−40.26	−31.92	80	120
DI water	1.33	−38.43	−34.05	89.75	40
Isopropanol	1.38	−44.2	−31.5	179.6	130
Methanol	1.33	−42.71	−31.98	110	134
Acetone	1.36	−38.89	−31.85	153	160
ETM	1.35	−39.76	−31.21	120	80
DII	1.36	−39.86	−31.2	120	120

## Abbreviations

The following abbreviations are used in this manuscript:

OMF	Optical microknot fiber
PR	Photonic resonance
SMF	Single-mode fiber
IR	Infrared
PDMS	Polydimethylsiloxane
FSR	Free spectral range
FWHM	Full width at half maximum
PS	Phase shift
ETM	Ethanol/Methanol mixture
DII	DI water/Isopropanol mixture

References
